# Super chickens, givers, and collective intelligence: the importance of collaboration, teamwork, and mentorship in science

**DOI:** 10.1172/JCI187403

**Published:** 2024-11-15

**Authors:** Benjamin D. Humphreys

**Affiliations:** Washington University, St. Louis, Missouri, USA

My wife Lisa is a teacher, and one rainy day last fall she was substitute teaching a new kindergarten class. Midmorning, one of her kindergartners earnestly asked, “Mrs. Humphreys, can we have indoor social joy today?” Confused, Lisa looked to her student teacher who took her aside to explain that “indoor social joy” is what they call recess in the gym during bad weather.

The Joint Meeting is “indoor social joy” for physician-scientists. Why? It is a unique chance to hear incredible talks, meet interesting and accomplished colleagues of all ages and interests, and even to strike up new collaborations. I want to talk a bit more about the importance of scientific collaborations.

I did not always appreciate how important collaborations are to moving science forward. I remember distinctly when a paper by Leonard Adleman, entitled, “Molecular computation of solutions to combinatorial problems,” came out in 1994 in the journal *Science* (1). Adleman was the sole author! I was a first-year graduate student at Case Western Reserve University, and I was just blown away by this first example of using DNA to perform computation. Adleman, a computer scientist at the University of Southern California, wanted to solve a complex problem — the Hamiltonian Path Problem. William Hamilton was Astronomer Royal for Ireland in the mid-19th century. The problem that bears his name is illustrated in Figure 1A. Let these arrows represent nonstop flights between cities. Some cities have only one nonstop whereas others have two or more. The problem is to determine if a path exists starting in city 0 and ending in city 6 while passing through each of the remaining cities only once.

In this simplified example (Figure 1B), the start city is Atlanta, and the end city is Detroit, and the Hamiltonian Path is Atlanta to Boston to Chicago to Detroit. The Hamiltonian Path Problem has been studied extensively by computer scientists, because no efficient algorithm has been devised to solve it. It is, in other words, computationally intense.

To use DNA to compute this Hamiltonian Path, Adleman designed unique oligos for each city, he also included their complement, and, finally, he devised flight paths that were a concatenation of the last half of the origin city and the first half of the destination city.

For example, Atlanta is represented by an eight-nucleotide sequence consisting of a unique four-nucleotide first name and a four-nucleotide last name. The flight path is encoded with the last name of the origin city and the first name of the destination city (Figure 2A). The last name of the destination city then becomes the new origin city for the next flight path and so on (Figure 2B). He then combined all these oligos in a test tube, added buffer and T4 DNA ligase, and in one second the reaction was done. Imagine, for example, this Atlanta to Boston flight path oligo annealing to the Boston complementary oligo, since the destination city is encoded by the first name. That leaves the complementary last name of Boston free to anneal with the next flight path, in this case Boston to Chicago (Figure 2C).

All possible combinations of ligations were created, however; so the challenge was to eliminate the 300 trillion wrong answers. He did this in an elegant way: (a) Eliminate any trips that do not start with Atlanta and end with Detroit, by performing PCR using forward primers complementary to Atlanta and backwards primers complementary to Detroit. (b) Eliminate any trips that had more or fewer than 4 stops, using gel electrophoresis and cutting out and saving only the band corresponding to 24-nucleotide oligonucleotides. (c) Ensure that remaining trips, which all had four stops and started in Atlanta and ended in Detroit, also passed through Boston and Chicago — by sequential affinity purification with oligos complementary to Boston, then complementary oligos for Detroit bound to magnetic beads.

In the end, the DNA had computed the correct Hamiltonian Path between the 7 cities. In interviews, Adleman described the “Aha moment” when this idea first came to him, at 1 am in bed. He was so excited he woke his wife up to tell her and then couldn’t go back to sleep.

To my first-year graduate student self, I thought that this was how science was done: A difficult problem suddenly solved elegantly in a moment of unexpected inspiration, while alone, possibly in an office surrounded by texts and diagrams, or perhaps, like Adleman, while in bed.

But of course, as I learned, that is not at all how scientific progress is made. In fact, that was not even the story for Adleman. It turns out that his idea of computing with DNA came to him only because he went on sabbatical in a molecular biology and virology lab the year before — where he was first introduced to the concept of complementary DNA, ligase, and DNA polymerase by other scientists (2).

I have subsequently come to understand that scientific progress is not simply improved by collaboration but requires it. Here is a personal and recent example. My undergraduate thesis concerned the writings of the great American author Herman Melville (Figure 3). You would not be mistaken in thinking that as an English Literature major, I never reached the level of math classes needed to understand these equations. Rather, these equations were generated by Nicolas Ledru, PhD, a talented WashU Medical Scientist Training Program student and computer scientist who finished his graduate work in my lab. For his thesis, Nicolas examined single-cell multiomic data to construct genome-wide parametric gene regulatory networks that generate predicted weights for both *cis*-regulatory elements and transcription factors to rank and prioritize the most important regulatory elements and, thereby, predict key drivers of cell state transitions (3). This was a highly successful exercise combining both computational and traditional wet lab approaches. I would like to think that part of the reason for the success of this project is that we both brought different and complementary skill sets to the project.

If scientific progress requires collaboration and teamwork, it surprises me that we do not think more in academic medicine about how to construct and nurture high-performing teams. It turns out there is a scientific literature on this topic. William Muir was an evolutionary biologist interested in productivity. He studied chickens because their productivity could be very easily measured — by simply counting their eggs they laid. He designed an elegant experiment. He took a group of average-producing chickens and combined them into a flock and bred them for six generations. He then took the highest-producing chickens he could find — you could call them super chickens — and combined them into a flock and bred them for six generations.

At the end of the experiment, the average chickens were healthy, had fluffy feathers, and were producing more eggs than when they started. For the super chickens, however, there were only three left, the rest having been pecked to death. These super chickens had only been high producers by suppressing the productivity of the other chickens around them (4).

I don’t know about you, but I have known a few super chickens during my time in academia.

This raises the point that simply bringing together highly driven overachievers can be counterproductive because of the negative effects of hypercompetitiveness on a group’s dynamic. By contrast, emphasizing collaboration over individual excellence can result in greater productivity. As another aside, I suspect we overrely on the super chicken model in deciding important leadership positions in academia.

What are other characteristics of high-performing teams? In a study out of the Massachusetts Institute of Technology, researchers brought 699 volunteers, put them in groups, and gave them very hard cognitive problems to solve (5). Not unexpectedly, some groups were far more successful than other groups. But the most successful groups were not those with the individuals who had the highest IQ, nor were they the groups that had the highest aggregate IQ. There were three characteristics of the most successful teams. First, they showed high social sensitivity to each other, as measured by the reading the mind in the eye test, which broadly measures empathy. Second, the most successful teams gave roughly equal time to each other. Nobody dominated and conversational turn-taking was distributed equally. Third, the most successful teams had more women. Whether this was because women score more highly on the reading the mind in the eye test or because women brought a more diverse perspective, we don’t know.

What this experiment shows, and I recommend this Ted Talk from Margaret Heffernan that describes it, is that more successful groups show higher social connectedness to each other (6). What happens between people really matters and leads to social capital — the reliance and interdependency that builds trust. This is only built with time together, leading to openness and candor. This creates a safe environment for conflict — which is required to turn a good idea into a great one. Think of lab meetings in collaborative and high-functioning labs where everyone asks questions and shares their ideas as a perfect example of this.

Adam Grant is an organizational psychologist at Penn who studies productivity in the workplace. He divides workers into three categories. Givers always support and help others with no strings attached. Takers are the opposite — they take as much as they can and give nothing in return. Most of us are matchers — we help others, but then expect the favor to be returned when we need something.

Grant studied productivity in the workplace — medical students’ grades, salespeople’s revenue, etc. — in 30,000 people across professions (7). He found that the worst performers across industries were the givers. They were so busy doing other people’s jobs that they ran out of time and energy to do their own. And yet, when examining the frequency of giving behavior in organizations, the higher the giving behavior, the better the organization performs on every measure of productivity that he could measure. Givers sacrifice themselves, but they make their organizations better.

So, who were the best performers? It is a relief to know that it is not the takers. They tend to have a fast rise and a fast fall. Why? Because the matchers soon find out that takers won’t reciprocate, and they make it a personal goal to punish the takers.

Are the best performers the matchers, then? It turns out the answer is no. The best performers are the givers. They represent both the worst performers and the best performers in a bimodal distribution. Grant asks how we can foster organizations where more of the givers get to excel. He has three answers — first, protect givers from burnout. Second, promote a culture of generosity and help-seeking behavior, because this tends to encourage giving behavior. Finally, keep out the takers. In a group dynamic, even one taker has a disproportionately negative effect on the group, causing givers to shut down.

Turning to scientific discovery, I want to make the point, primarily for the younger people in the audience, that we are in a remarkable Golden Age of medicine and scientific discovery (8). Nobel Prize winner Jennifer Doudna said, “I’ve been running my research lab for almost 30 years. And I can say that throughout that period of time, I’ve just never experienced what we’re seeing just over the last five years.” Think about it — we have CAR T therapy, genome editing, big data, Glp-1 agonists, SGLT2 inhibitors, and artificial intelligence. Two patients have been transplanted with genetically modified pig kidneys this year. These are incredibly exciting times, and I wish I was just starting my career because the next 50 years will be truly remarkable.

But my last point is that no matter how exciting scientific discovery is, and believe me I love it, in my midcareer I really came to believe that, personally, my students and trainees are my legacy (9). In 1974, Studs Terkel published a book called *Working: People Talk About What They Do All Day and How They Feel About What They Do* (10). He interviewed in detail over a hundred people in different jobs. Barbers, waitresses, doctors, teachers, and so on. In short, it is an exploration of the meaning of work. Two themes emerged: workers in general were happy with their careers if they built something lasting, like the Golden Gate Bridge, or if they helped to shape people’s lives (for example, patients or parishioners). Along these lines, then, I do believe that my long-lasting impact will be people, not papers, despite deriving great satisfaction from scientific investigation.

It is in that spirit that I am very happy and proud to report that over the last year we on the ASCI Council and staff have worked to create the ASCI Postbac Program. This is a two-year experience in which we will match two Postbacs into two ASCI member labs. We will provide a full stipend, partner with the APSA to provide them with mentors who are still in training, provide MCAT test prep, counseling on medical school essays, and bring them to the Joint Meeting. Primarily, they will have an intensive, mentored lab experience with a current MSTP or PSTP in the lab that will expose them to the physician-scientist career pathway and strengthen their applications to MD or MD/PhD programs. We had a very strong group of applicants in this our first year and successfully matched two outstanding Postbacs to ASCI member laboratories.

We have committed funding for 5 years and will track outcomes. It is our sincere hope that we can expand this program, and my call to action today is for those of you who can, either personally or through your company, partner with us so we can expand this program and continue it into the future in order to support the fragile physician-scientist pipeline.

In conclusion, scientific progress requires collaboration. Be a giver or support them. Your students are your legacy.

I have many people to thank. Karen Guth and John Hawley are the heart and soul of the ASCI, and they have been just incredible to work with over the last six years, as have the dedicated staff, Maya Hoptman, Eileen Rojas, Sarah Jackson, Colleen McGarry, Theresa Kaiser, and Corinne Williams.

The ASCI Council is an incredibly talented and collegial group. Each fall we spend two days selecting new members followed by an activity in a warm place. Last year was not so warm, and we were a bit of a motley crew, but Boneyard Beach north of Charleston was dramatic (Figure 4).

I have had the great fortune to work very closely with my friends Past-President Sohail Tavazoie and soon-to-be President Anna Greka. They have each provided wise counsel, unwavering commitment, and visionary leadership, and I owe them both a deep debt of gratitude.

I also want to recognize two of my own role models and mentors. My father Michael Humphreys, now retired, spent his career also as a nephrologist at San Francisco General, a safety-net hospital. My mother Sheila Humphreys spent her career at the University of California, Berkeley, in the Electrical Engineering and Computer Science Department where she was their inaugural Director of Diversity from 1982 to 2015 (11). Her advocacy and mentorship were recognized by President Obama with a Presidential Award for Excellence in Science, Mathematics, and Engineering Mentoring.

And finally, a huge thanks to my family. My wife Lisa is here today, and our three children are Sam, Peter, and Wendy (Figure 5). They are an enormous source of pride and support. They also keep me grounded. Our daughter Wendy is a sophomore at the University of Rochester where she is a computer science major — a woman in STEM. Back when she was five years old, I went to kiss her goodnight and overheard her say to my wife, “Mommy, you are the sweetest thing in the world.”

Lisa replied, “No Wendy, you are the sweetest thing in the world.”

“We are both the sweetest thing, Mommy,” Wendy responded.

Standing there, I asked tentatively, “What am I?”

There was the slightest pause, and Wendy said, “Daddy, you are the Pumpkinhead.”

In conclusion, serving as your President Pumpkinhead has been the singular honor and privilege of my professional career. Thank you.

## Figures and Tables

**Figure 1:**
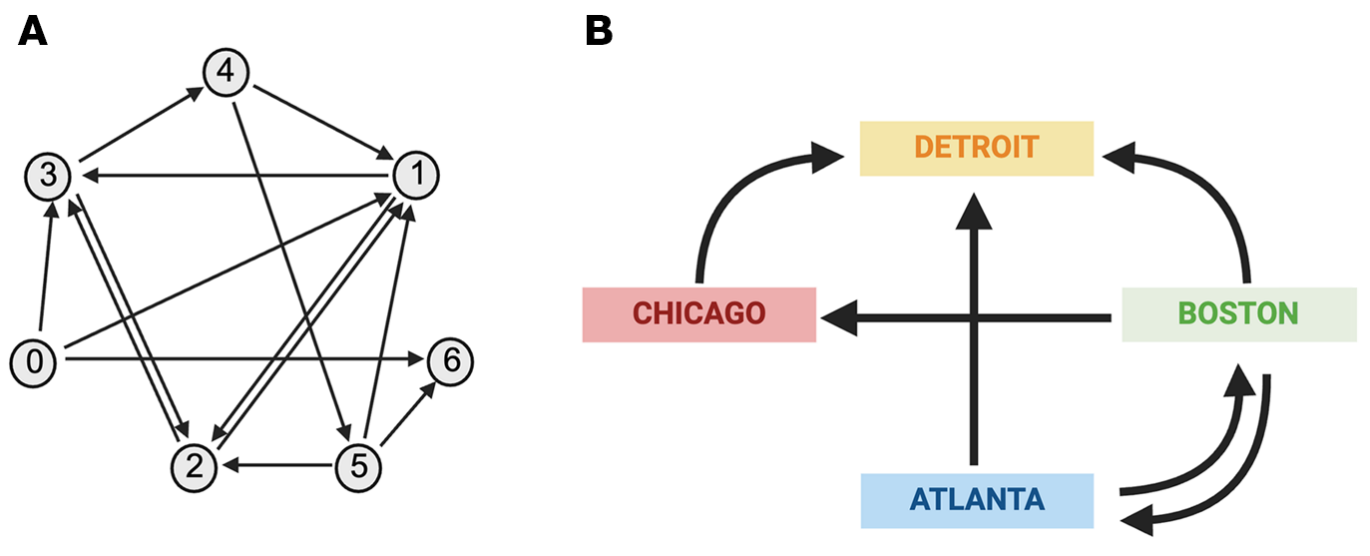
The Hamiltonian Path Problem. (**A**) The unique Hamiltonian path in this example is 0 → 1, 1 → 2, 2 → 3, 3 → 4, 4 → 5, 5 → 6. Adapted from ref. 12. Adapted from *Scientific American* with permission from Andy Christie. (**B**) A simplified Hamiltonian Path with four cities, where arrows represent flights between the cities. The Hamiltonian Path in this example is Atlanta → Boston → Chicago → Detroit. Created in BioRender. Adapted with permission from *Nature* (12).

**Figure 2:**
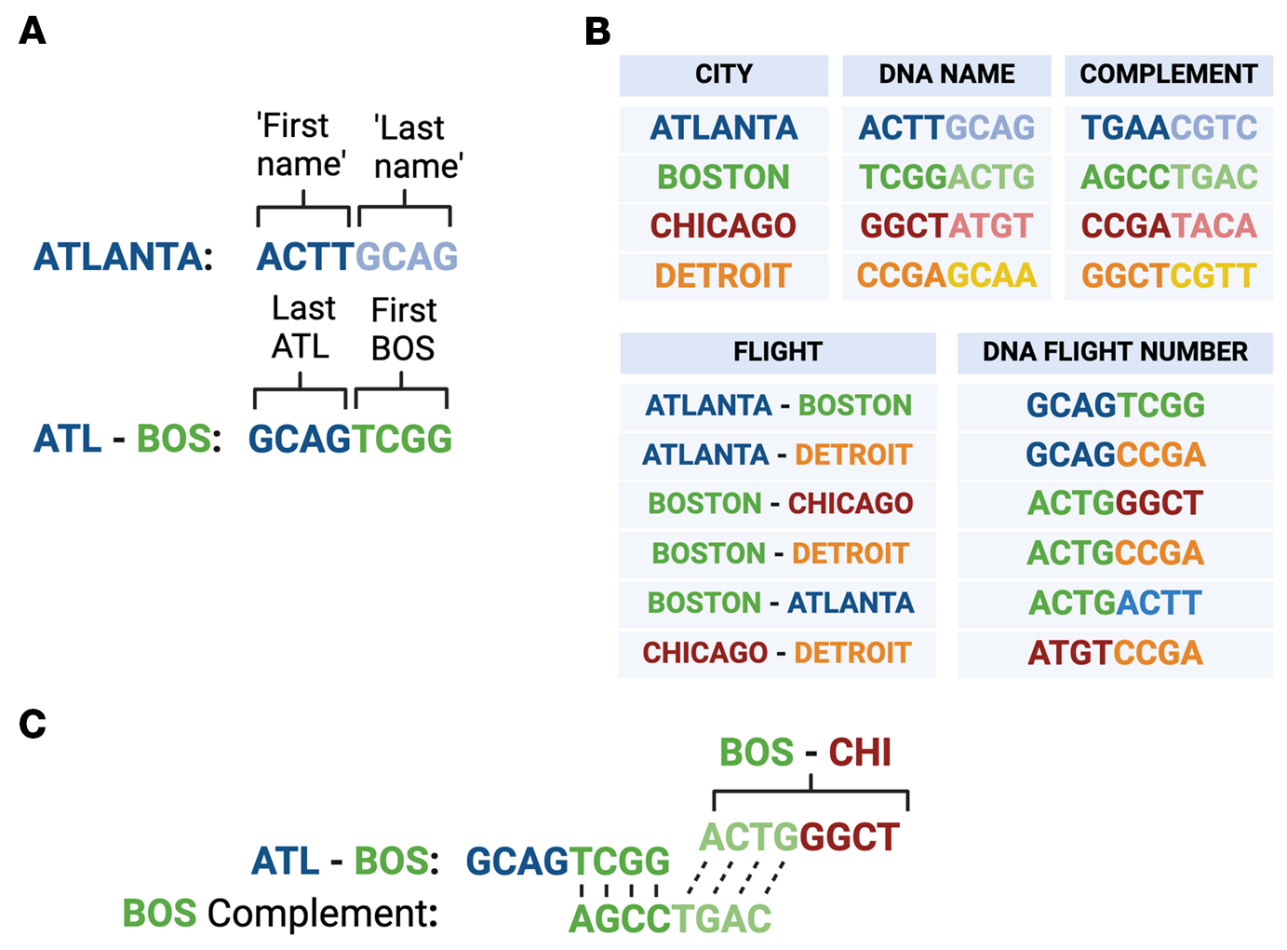
Design of oligonucleotide cities and flight paths. (**A**) Encoding of each city and possible flightpath by a unique eight-oligonucleotide sequence. (**B**) City oligonucleotides and their complement and DNA flight numbers represent possible flights between cities. (**C**) Example of how a flight path from Atlanta to Boston to Chicago would be calculated through oligonucleotide annealing. Adapted with permission from *Nature* (12). Created in BioRender.

**Figure 3:**
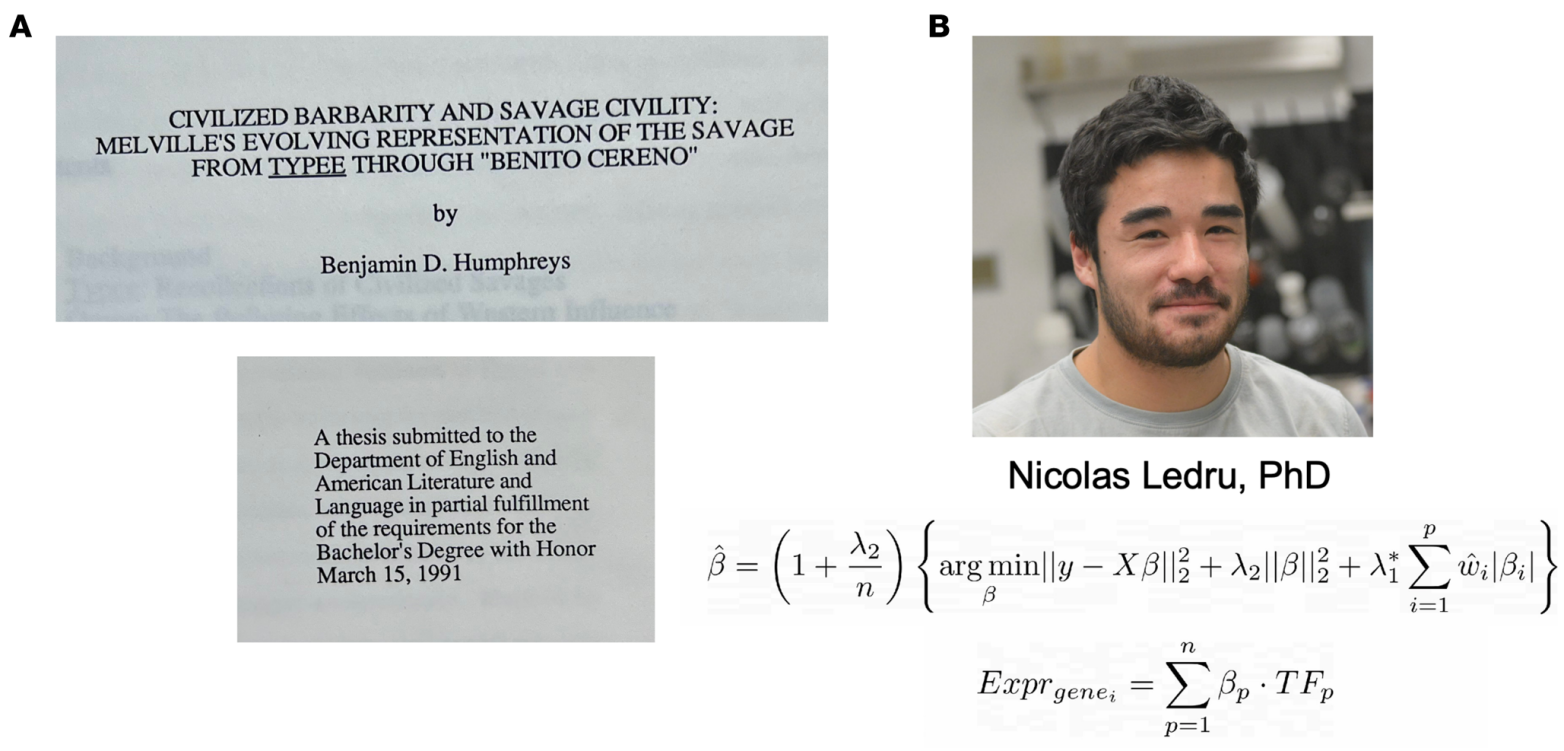
(**A**) The author’s undergraduate thesis. (**B**) Nicolas Ledru, PhD, a Washington University medical scientist training program student.

**Figure 4:**
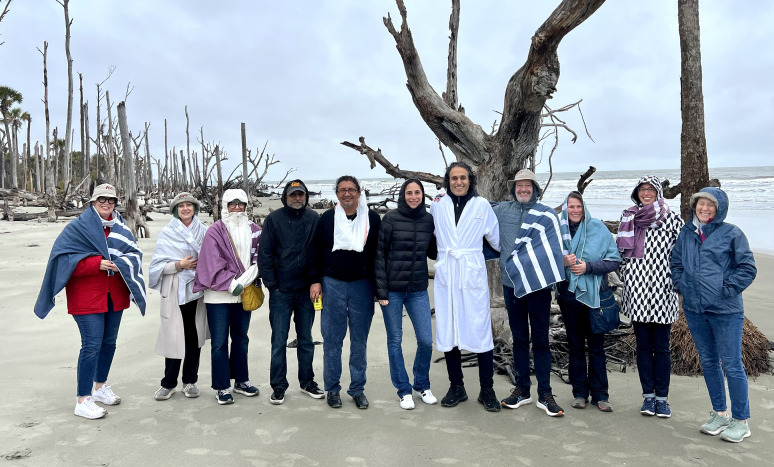
The ASCI Council and staff at Boneyard Beach, South Carolina, in 2023.

**Figure 5:**
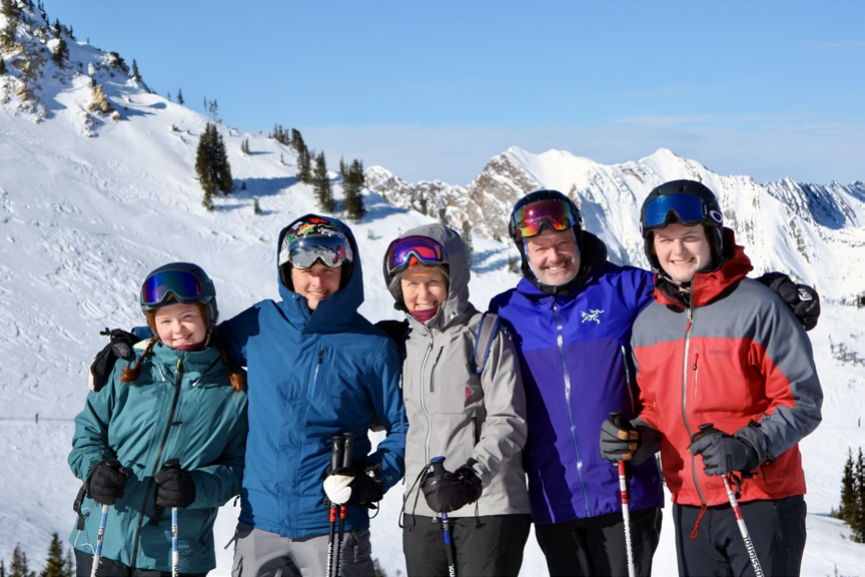
The Humphreys family, 2023. Left to right: Wendy, Sam, Lisa, Ben, and Peter.

## Version 1 11/15/2024

Electronic publication
